# Associations of Household Expenditure and Marital Status With Cardiovascular Risk Factors in Japanese Adults: Analysis of Nationally Representative Surveys

**DOI:** 10.2188/jea.JE20120021

**Published:** 2013-01-05

**Authors:** Yoshiharu Fukuda, Ayako Hiyoshi

**Affiliations:** 1 Department of Community Health and Medicine, Yamaguchi University School of Medicine, Ube, Yamaguchi, Japan; 1 山口大学医学部地域医療推進学講座; 2 Department of Epidemiology and Public Health, University College London, London, UK; 2 University College London-Department of Epidemiology and Public Health

**Keywords:** health inequalities, socioeconomic factor, household expenditure, cardiovascular risk factor, marital status

## Abstract

**Background:**

Socioeconomic inequalities in health and social determinants of health are important issues in public health and health policy. We investigated associations of cardiovascular risk factors with household expenditure (as an indicator of socioeconomic status) and marital status in Japan.

**Methods:**

We combined data from 2 nationally representative surveys—the Comprehensive Survey of Living Conditions and the National Health and Nutrition Survey, 2003–2007—and analyzed sex-specific associations of household expenditure quartiles and marital status with cardiovascular risk factors, including obesity, hypertension, dyslipidemia, and diabetes, among 6326 Japanese adults (2664 men and 3662 women) aged 40 to 64 years.

**Results:**

For men, there was no statistically significant association between household expenditure and cardiovascular risk factors. For women, lower household expenditure was significantly associated with obesity, hypertension, diabetes, and the presence of multiple risk factors: the ORs for the lowest versus the highest quartile ranged from 1.39 to 1.71. In a comparison of married and unmarried participants, the prevalence of cardiovascular risk factors was higher among married women and lower among married men.

**Conclusions:**

Lower socioeconomic status, as indicated by household expenditure, was associated with cardiovascular risk factors in Japanese women. Socioeconomic factors should be considered in health promotion and prevention of cardiovascular disease.

## Abstract (ja)

個人の社会経済的な状況によって健康水準が異なることが知られており、健康格差や健康の社会的決定要因が公衆衛生や健康政策で重要になっている。この研究は、社会経済的要因として家計支出と婚姻状況に注目し、これらと循環器疾患のリスク要因との関連について分析を行った。調査は、国の代表的な調査である国民生活基礎調査と国民健康・栄養調査（平成15～19年）に参加した40歳から64歳の男性2664名と女性3662名のデータを用いた。家計支出ならびに婚姻状況によって、循環器疾患のリスク要因である肥満、高血圧、脂質異常症、糖尿病の割合が異なるかを解析した。男性では、家計支出とリスク要因とに有意な関係は認められなかった。一方、女性では、家計支出が低いほど、肥満、高血圧、糖尿病、メタボリックシンドロームに準じた複数のリスクを持つ者の割合が高かった。女性において、最も家計支出の高い群に比較した最も低い群のオッズ比は1.39から1.71だった。婚姻状態との関連では、女性では既婚者は未婚者よりリスク要因の割合が高くなっていたが、男性では逆の関係が認められた。この研究では、女性でのみ家計支出は循環器疾患のリスク要因と関連していた。社会経済的に好ましくない状況にある者ほど、循環器疾患になりやすくなる可能性が示唆されたが、性別や婚姻状態によりその関連性は異なった。健康づくりや循環器疾患の予防において社会経済的な要因にも考慮しなければならないことが示された。

## INTRODUCTION

Socioeconomic inequalities in health are important concerns in public health and health policy. Health is determined by a wide range of indicators of individual socioeconomic status and social environment, which are referred to as social determinants of health.^1^ Social epidemiology is the branch of epidemiology that examines health inequalities and social determinants of health.^2^ Social epidemiologic studies of the Japanese population, which followed such studies in Western countries, have been conducted since the late 1990s^3^ and have revealed disparities in mortality, morbidity, self-rated health, psychological distress, health behaviors, and other health outcomes in relation to indicators of socioeconomic status such as educational attainment, income, and occupational class.^3^

Cardiovascular disease, including stroke and ischemic disease, is one of the most highly prioritized health problems in Japan and other industrialized countries. Many social epidemiologic studies in Western countries have noted associations of cardiovascular disease and its risk factors with socioeconomic factors.^4^^–^^6^ Income is one of the most commonly used socioeconomic indicators and has a strong relationship with cardiovascular risk factors.^5^^,^^7^^–^^9^ In Japan, there have been several studies of the association of cardiovascular disease and its risk factors with education and occupational class.^10^^–^^13^ However, there is no systematic analysis of the association between income and cardiovascular risk factors in the Japanese population. The present study used household expenditure as a surrogate indicator of household economy. Although the relationship between household expenditure and health status is rarely studied, the value of such data has been demonstrated.^14^

We also examined the association with marital status, an important social determinant of health. Adults who are single, separated, or divorced have higher mortality in most countries, including Japan.^15^^–^^17^ However, only a few studies examined the association between marital status and cardiovascular risk factors, and the findings were not conclusive.^18^^,^^19^

Disparities in education, income, and employment in the Japanese population are receiving more attention^20^; thus, it is necessary to obtain reliable evidence and monitor socioeconomic inequalities in health throughout Japan. For these purposes, analyses of data from nationally representative surveys are effective. A current national survey in Japan showed a clear inverse relationship between household income and the prevalences of obesity and smoking.^21^ In addition, accumulating evidence on the association between cardiovascular risk factors and socioeconomic status suggests the importance of socioeconomic factors in preventing cardiovascular disease.

In the present study, we used data from 2 nationally representative surveys: the Comprehensive Survey of Living Conditions (CSLC) and the National Health and Nutritional Survey (NHNS).^22^^,^^23^ CSLC collected a wide range of information such as demographic characteristics, household expenditure, and employment status. NHNS includes objective health measurements such as anthropometric and laboratory data. We linked these 2 datasets to examine the association of household expenditure, as an index of socioeconomic status, and marital status with cardiovascular risk factors, including obesity, hypertension, diabetes, and dyslipidemia in Japan.

## METHODS

### Data

We used data from CSLC and NHNS, 2003–2007, which were conducted by the Ministry of Health, Labour and Welfare in Japan.^22^^,^^23^ CSLC began in 1986 and has conducted large surveys every 3 years and small surveys in the years between. For all surveys, the entire land area of Japan was divided into approximately 1 million enumeration districts (EDs). The large surveys randomly selected approximately 5000 EDs, while the small surveys selected 1000 EDs. Using lists of households, interviewers visited all households within the selected areas and approached all household members. The questionnaires included basic household and individual information on demographics, health, illness profile, lifestyle, monthly household expenditure, and other items. The numbers of households approached were 55 307, 276 682, 56 125, 58 251, and 287 807 from 2003 to 2007. The response rate ranged from 79.9% in 2004 to 81.6% in 2007 (average 80.2%).^23^ Ultimately, 1 613 784 individuals from 587 653 households in 13 880 EDs were included in the analysis.

NHNS is an annual nationwide nutrition survey that began in 1948. Using the EDs of CSLC, 300 EDs were randomly selected every year, and all household members older than 1 year were approached. The exact number of participants approached was not published. The approximate annual number of participants in 2003–2007 was 15 000 household members from 5000 households. The household-based response rates ranged from about 60% in 2007 to 83% in 2003, and response rates varied by survey item.^22^ We ultimately obtained data from 50 209 individuals.

NHNS comprised (1) an anthropometric examination including height, weight, and blood pressure (BP); (2) blood testing including triglyceride (TG), high-density lipoprotein cholesterol (HDL-C), fasting blood sugar (FBS), and hemoglobin A_1_c (HbA_1_c); (3) a lifestyle survey including smoking habit; and (4) a 1-day semi-weighed dietary record. Height and weight were measured with participants in light clothing without shoes. Body mass index (BMI) was calculated as the ratio of weight in kilograms to the square of the height in meters. The mean of 2 measurements of systolic and diastolic BP was calculated. Using a standard mercury sphygmomanometer, the first BP measurement was taken on the right arm after a minimum 5-minute rest, with the participant in sitting position. The second measurement was taken 1 to 2 minutes after the first measurement. After a minimum 4-hour fast, blood samples were collected from participants through the antecubital vein. TG, HDL-C, and FBS were analyzed using the H7170 (Hitachi, Japan), and HbA_1_c was analyzed using the BM-9030 (JEOL, Japan). Details are available on the survey website.^22^

The procedure for selecting the participants is shown in Figure. Because NHNS and CSLC share sampling units, we were able to link the survey datasets, using survey year, prefecture, area, household number, number of household members, sex, and age. Among 50 209 participants of NHNS, 49 509 were linked. Then, we excluded participants who were younger than 39 years or older than 65 years and those with missing data on household expenditure or other variables analyzed in this study. Ultimately, data from 6326 participants (2664 men and 3662 women) were analyzed. The data from the 2 surveys were used with permission from the Japanese Ministry of Health, Labour and Welfare.

**Figure.**
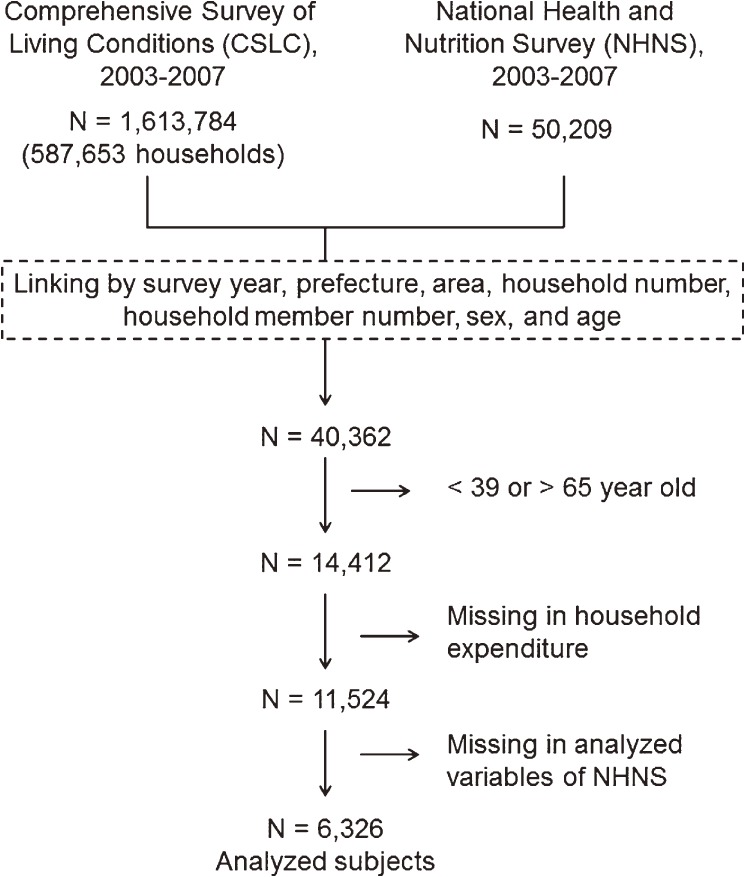
Procedure for selecting participants

### Outcomes

The outcome variables were cardiovascular risk factors, ie, obesity, hypertension, dyslipidemia, diabetes, presence of multiple risk factors, and current smoking. The definitions of these conditions corresponded to the criteria of the National Health Program.^24^ Obesity was defined as a BMI of at least 25 kg/m^2^; hypertension as systolic BP of at least 140 mm Hg and/or diastolic BP of at least 90 mm Hg; high TG as a level of at least 300 mg/dl for men or at least 150 mg/dl for women; low HDL-C as a level less than 35 mg/dl for men or less than 40 mg/dl for women; high FBS as a level of at least 126 mg/dl; high HbA_1_c as a level of at least 6.1% (Japan Diabetes Society standard); and presence of multiple risk factors as obesity plus 2 or more risk factors among hypertension, dyslipidemia (high TG and/or low HDL-C), and diabetes (high FBS and/or high HbA_1_c). Current smoking was defined as (1) a history of smoking more than 100 cigarettes or smoking for longer than 6 months and (2) currently smoking every day or occasionally.

### Analyses

Equivalent household expenditure was used as a socioeconomic measure and was calculated by dividing household expenditure per month by the square root of household size, according to the recent method of the Organisation for Economic Cooperation and Development (OECD).^25^ The study participants were then grouped by sex and quartile of household expenditure. Table 1 shows the quartiles of household expenditure according to participant age and marital status.

**Table 1.** Characteristics of household expenditure quartiles, by participant age and marital status

	Quartile	*n*	Monthly household expenditure (×1000 yen)				Age, y	Marital status

			Min	Max	Median	Mean	(Mean ± SD)	(% married)
Men	1st (lowest)	666	11.5	105.0	80.8	77.6	53.6 ± 7.3	79.4%
	2nd	686	105.8	141.4	125.0	124.3	54.5 ± 7.3	90.5%
	3rd	642	142.9	198.0	165.0	164.9	54.0 ± 6.8	91.4%
	4th (highest)	670	200.0	6364.0	258.3	372.4	54.8 ± 6.7	87.5%

Women	1st (lowest)	921	7.1	106.1	86.6	82.2	53.9 ± 7.2	74.6%
	2nd	918	106.1	144.3	130.0	129.4	53.1 ± 7.4	86.6%
	3rd	921	145.0	200.0	173.2	171.0	52.7 ± 7.1	87.7%
	4th (highest)	902	201.2	6364.0	250.0	372.4	53.7 ± 7.1	90.4%

Statistical analyses were conducted separately for men and women. The prevalences of cardiovascular risk factors were calculated with respect to household expenditure quartile and marital status. The odds ratios (ORs) of household expenditure quartiles versus the fourth (the highest) quartile and marital status (being married vs being unmarried, including separated and divorced) were computed using multiple logistic regression analysis. Each model included household expenditure quartile, marital status, and age as explanatory variables. Analysis of trends in the association between household expenditure and risk factors was done using models with household expenditure quartiles as a continuous variable. The statistical package SPSS 19.0 (IBM, Ireland) was used for all statistical analyses.

## RESULTS

Table 2 shows selected characteristics of the participants, including the prevalences of cardiovascular risk factors. Prevalence ranged from 3.9% (for low HDL-C) to 44.8% (for current smoking) among men and from 2.7% (for low HDL-C) to 30.6% (for hypertension) among women.

**Table 2.** Selected characteristics and summary of cardiovascular risk factors of study participants

Variables	Criteria	Men	Women
		(*n* = 2664)	(*n* = 3662)
Age (y)		54.2 ± 7.1	53.4 ± 7.1
Married		87.2%	84.8%
Obesity	Body mass index ≥25 kg/m^2^	34.6%	22.1%
Hypertension	Systolic blood pressure ≥140 mm Hg and/or diastolic blood pressure ≥90 mm Hg	45.9%	30.6%
High triglyceride (TG)	≥300 mg/dl (men); ≥150 mg/dl (women)	9.6%	25.1%
Low HDL cholesterol (HDL-C)	<35 mg/dl (men); <40 g/dl (women)	3.9%	2.7%
High fasting blood sugar (FBS)	≥126 mg/dl	14.4%	9.5%
High hemoglobin A_1_c (HbA_1_c)	≥6.1%	10.5%	6.2%
Multiple risk factors	Obesity and ≥2 other factors among hypertension, dyslipidemia,^a^ and diabetes^b^	31.9%	18.6%
Current smoking		44.8%	10.5%

^a^High TG and/or low HDL-C.

^b^High FBS and/or high HbA_1_c.

The association of cardiovascular risk factors with household expenditure quartile and marital status among men is shown in Table 3. A significantly higher OR, as compared with the fourth (highest) quartile, was found only in the second quartile for low HDL-C. There was no significant trend in relation to household expenditure for any risk factor. Regarding marital status, married men were less likely to have hypertension, high FBS, high HbA_1_c, and multiple risk factors.

**Table 3.** Association of household expenditure with cardiovascular risk factors among men

Explanatory variables	Obesity			Hypertension			High triglyceride			Low HDL cholesterol		
			
	%	OR	(95% CI)	%	OR	(95% CI)	%	OR	(95% CI)	%	OR	(95% CI)
Household expenditure quartile												
1st (lowest)	36.3	1.04	(0.83–1.30)	46.1	1.09	(0.88–1.36)	9.5	0.86	(0.60–1.24)	2.4	0.72	(0.37–1.39)
2nd	35.1	1.01	(0.81–1.26)	46.4	1.09	(0.88–1.35)	9.8	0.94	(0.66–1.35)	6.1	1.94	(1.14–3.29)
3rd	31.8	0.87	(0.69–1.09)	46.6	1.13	(0.90–1.40)	8.7	0.83	(0.57–1.20)	3.7	1.16	(0.64–2.10)
4th (highest)	35.1	1.00	(reference)	44.8	1.00	(reference)	10.3	1.00	(reference)	3.3	1.00	(reference)
*P* for trend	0.497			0.508			0.569			0.968		
Marital status												
Married	34.0	0.84	(0.66–1.07)	45.4	0.78	(0.62–0.99)	9.2	0.74	(0.52–1.06)	3.9	0.82	(0.46–1.48)
Not married	38.7	1.00	(reference)	49.9	1.00	(reference)	12.3	1.00	(reference)	4.1	1.00	(reference)

Explanatory variables	High fasting blood sugar			High hemoglobin A_1_c			Multiple risk factors			Current smoking		
			
	%	OR	(95% CI)	%	OR	(95% CI)	%	OR	(95% CI)	%	OR	(95% CI)
Household expenditure quartile												
1st (lowest)	13.1	0.92	(0.77–1.29)	9.0	0.79	(0.55–1.14)	32.4	0.97	(0.77–1.22)	46.9	1.18	(0.95–1.48)
2nd	16.6	1.25	(0.93–1.69)	11.5	1.06	(0.75–1.48)	33.4	1.04	(0.83–1.31)	44.5	1.12	(0.90–1.39)
3rd	13.9	1.04	(0.76–1.42)	10.1	0.95	(0.67–1.36)	29.0	0.95	(0.67–1.07)	46.5	1.19	(0.96–1.49)
4th (highest)	14.0	1.00	(reference)	11.2	1.00	(reference)	32.7	1.00	(reference)	41.5	1.00	(reference)
*P* for trend	0.951			0.341			0.788			0.207		
Marital status												
Married	13.7	0.61	(0.45–0.83)	10.0	0.63	(0.44–0.89)	31.2	0.78	(0.61–0.99)	44.4	0.93	(0.73–1.17)
Not married	19.1	1.00	(reference)	13.5	1.00	(reference)	37.0	1.00	(reference)	47.6	1.00	(reference)

ORs were estimated using a logistic regression model that included household expenditure, marital status, and age.

*P* for trend was estimated using a model in which household expenditure quartile was represented as a continuous variable.

For women, as shown in Table 4, the trend in relation to household expenditure was significant for obesity, high FBS, and presence of multiple risk factors. A significantly higher OR for the first (lowest) versus the fourth (highest) quartile was observed for obesity, hypertension, high FBS, high HbA_1_c, and presence of multiple risk factors. ORs ranged from 1.39 (95% CI:1.13–1.71) for hypertension to 1.71 (1.23–2.37) for high FBS. Regarding hypertension, a significantly higher OR was found in the first and third quintiles but not in the second quintile. Although the rate of current smoking increased with decreasing household expenditure, ORs were not significant after adjustment for age and marital status. Married women were more likely to be obese and to have hypertension and multiple risk factors and less likely to be current smokers.

**Table 4.** Association of household expenditure with cardiovascular risk factors among women

Explanatory variables	Obesity			Hypertension			High triglyceride			Low HDL cholesterol		
			
	%	OR	(95% CI)	%	OR	(95% CI)	%	OR	(95% CI)	%	OR	(95% CI)
Household expenditure quartile												
1st quartile (lowest)	27.3	1.69	(1.35–2.12)	33.9	1.39	(1.13–1.71)	24.8	1.05	(0.84–1.30)	3.1	1.28	(0.72–2.26)
2nd quartile	23.4	1.37	(1.09–1.72)	31.3	1.27	(1.03–1.56)	25.8	1.14	(0.92–1.41)	2.2	0.91	(0.49–1.67)
3rd quartile	19.1	1.06	(0.84–1.34)	29.6	1.21	(0.98–1.50)	26.1	1.17	(0.95–1.46)	3.1	1.35	(0.77–2.37)
4th quartile (highest)	18.6	1.00	(reference)	27.5	1.00	(reference)	23.8	1.00	(reference)	2.4	1.00	(reference)
*P* for trend	<0.001			0.002			0.753			0.711		
Marital status												
Married	22.4	1.27	(1.01–1.60)	30.8	1.26	(1.03–1.55)	25.1	1.05	(0.85–1.30)	2.7	0.97	(0.56–1.66)
Not married	20.5	1.00	(reference)	29.6	1.00	(reference)	25.3	1.00	(reference)	3.1	1.00	(reference)

Explanatory variables	High fasting blood sugar			High hemoglobin A_1_c			Multiple risk factors			Current smoking		
			
	%	OR	(95% CI)	%	OR	(95% CI)	%	OR	(95% CI)	%	OR	(95% CI)
Household expenditure quartile												
1st quartile (lowest)	11.7	1.71	(1.23–2.38)	7.3	1.51	(1.01–2.25)	22.8	1.69	(1.32–2.13)	13.0	1.26	(0.93–1.72)
2nd quartile	9.8	1.45	(1.04–2.04)	6.3	1.38	(0.92–2.08)	19.7	1.37	(1.07–1.75)	10.0	1.02	(0.74–1.40)
3rd quartile	9.2	1.42	(1.01–1.99)	6.3	1.44	(0.95–2.16)	16.3	1.11	(0.85–1.41)	9.8	1.00	(0.73–1.38)
4th quartile (highest)	7.2	1.00	(reference)	4.8	1.00	(reference)	15.6	1.00	(reference)	9.1	1.00	(reference)
*P* for trend	0.002			0.075			<0.001			0.132		
Marital status												
Married	9.4	1.17	(0.85–1.59)	6.0	0.95	(0.66–1.36)	19.1	1.42	(1.10–1.82)	8.9	0.40	(0.31–0.51)
Not married	9.9	1.00	(reference)	7.4	1.00	(reference)	16.2	1.00	(reference)	19.2	1.00	(reference)

ORs were estimated using a logistic regression model that included household expenditure, marital status, and age.

*P* for trend was estimated using a model in which household expenditure quartile was represented as a continuous variable.

## DISCUSSION

Using the data from 2 nationally representative surveys, this study examined the associations of household expenditure and marital status with cardiovascular risk factors in the Japanese population. Lower household expenditure was associated with an increase in risk factors among women but not men. In addition, there were sex differences in the association with marital status.

Because income and household expenditure are rarely measured in health surveys in Japan, there are limited data on income-related inequalities in cardiovascular risk factors, especially hypertension, diabetes, and dyslipidemia. Our previous study showed that lower income was associated with increased morbidity from these diseases; however, the data were self-reported.^26^ Other studies have examined cardiovascular risk factors in relation to education and occupation,^12^^,^^13^^,^^27^ but the results were not consistent. One such study showed that education level was inversely associated with BMI among female civil servants.^27^ In contrast, a study of male company workers found a positive association between employment grade and BMI.^12^ Although some studies reported that hypertension, diabetes, and dyslipidemia are associated with education, the results have been inconsistent.^12^^,^^13^ The use of a national sample with objective measurements enabled us to collect strong evidence of socioeconomic inequalities in cardiovascular risk factors: lower socioeconomic status, as measured by household expenditure, was associated with increased obesity, hypertension, and diabetes among women.

The sex difference in the association between household expenditure and cardiovascular risk factors is an interesting finding of this study. Prior Japanese ecologic studies showed that the relationship between socioeconomic status and some health measurements was stronger in men than in women.^28^^,^^29^ However, findings from individual-based studies have not consistently shown such sex differences.^10^^,^^11^^,^^13^^,^^27^ Other studies using national samples found that socioeconomic differences in smoking and dietary habits were larger in women than in men.^30^^,^^31^ In Western countries, the relationship between socioeconomic status and cardiovascular risk factor was stronger among women than men.^32^^–^^35^ These sex differences might be related to parity, the stigma of obesity, and psychosocial risks.^32^^,^^34^ First, childbirth is associated with increased central obesity and decreased HDL-C, and education level is inversely associated with number of childbirths. Second, the stigma of obesity might be stronger in women than men, and obesity might be more likely to hamper upward social mobility in women. Last, being in low socioeconomic strata may cause additional psychological burdens for women. Further research is necessary to investigate whether these explanations account for sex differences in cardiovascular risk factors in Japan.

Another interesting sex difference in this study was the association between marital status and cardiovascular risk factors. Being married was associated with lower prevalence of cardiovascular risk factors among men and thus might be protective against cardiovascular disease. However, among women, being married was associated higher prevalences of obesity, hypertension, and multiple risk factors. In prior studies of marital status and health behavior in Japan, the association differed by type of behavior, the details of marital status, and age.^30^^,^^31^ Further studies are necessary to evaluate the effect of marital status on cardiovascular risk factors and health behavior, including its mediating and modifying roles in socioeconomic differences in mortality.

The most important limitation of this study is the use of household expenditure as an indicator of socioeconomic status. Because household income was shown to be more sensitive than expenditure, it might be a more appropriate indicator of socioeconomic status. Unfortunately, income information on CSLC was not collected for NHNS participants in the survey years of this study. Therefore, we used the equivalent, household expenditure, which has been demonstrated to be useful for social patterning of risk factors.^14^ NHNS has included income information since 2010, and the associations of obesity and smoking with household income was evident.^21^ In comparison to the analysis using income, the association with expenditure in the present study was moderate. Therefore, the present results are likely to be conservative estimates of socioeconomic differences.

There are other important limitations. First, because we used household expenditure as an explanatory variable and adjusted only for age and marital status, other likely confounding and mediating factors such as education, occupational class, and region of residence were not considered. Second, although the study samples were randomly selected, the response rates were not very high, particularly for NHNS. If participants with lower household expenditure were less likely to respond, the relationship between household expenditure and cardiovascular risk factors might have been underestimated. Third, the analyzed participants were a very small sample of the participants of CSLC and NHNS, which might have induced selection bias. Finally, the analyzed participants were limited to the age group 40 to 65 years. Socioeconomic inequalities in health differ by age group^29^^,^^30^; thus, it might not be appropriate to extrapolate our results to other age groups.

The present findings emphasize the importance of socioeconomic factors in health promotion and prevention of cardiovascular disease. Such programs should focus more on socioeconomically disadvantaged populations. A previous study reported that Japanese mistakenly believed that risk factors such as obesity, hypertension, and diabetes were more prevalent among people of higher socioeconomic status.^36^ The fact that these risk factors are associated with lower socioeconomic status needs to recognized by the population.

In conclusion, using data from 2 nationally representative surveys, we found that lower household expenditure was associated with cardiovascular risk factors, including obesity, hypertension, and diabetes, among women but not men. Although the pathways from lower household expenditure to these risk factors were not within the scope of this study, these associations might result in socioeconomic inequalities in mortality. Socioeconomic factors should be considered in health promotion and prevention of cardiovascular disease.

## ONLINE ONLY MATERIALS

The Japanese-language abstract for articles can be accessed by clicking on the tab labeled Supplementary materials at the journal website http://dx.doi.org/10.2188/jea.JE20120021.

Abstract in Japanese.
