# Supplementary material for: Associations of Household Expenditure and Marital Status With Cardiovascular Risk Factors in Japanese Adults: Analysis of Nationally Representative Surveys
Source: J Epidemiol. 2013 Jan 5;23(1):21–7. doi: 10.2188/jea.JE20120021 (PMC3700239; doi:10.2188/jea.JE20120021)
Supplement: Abstract in Japanese. [file je-23-021-s001.pdf]

日本人成人における家計支出および婚姻状況と循環器疾患のリスク要因の関連

福田吉治[1]、日吉綾子[2]

[1] 山口大学医学部地域医療推進学講座、[2]University College London-Department of Epidemiology and Public Health

個人の社会経済的な状況によって健康水準が異なることが知られており、健康格差や健康の社会的決定要因が公衆衛生や健康政策で重要になっている。この研究は、社会経済的要因として家計支出と婚姻状況に注目し、これらと循環器疾患のリスク要因との関連について分析を行った。調査は、国の代表的な調査である国民生活基礎調査と国民健康・栄養調査（平成15～19年）に参加した40歳から64歳の男性2664名と女性3662名のデータを用いた。家計支出ならびに婚姻状況によって、循環器疾患のリスク要因である肥満、高血圧、脂質異常症、糖尿病の割合が異なるかを解析した。男性では、家計支出とリスク要因とに有意な関係は認められなかった。一方、女性では、家計支出が低いほど、肥満、高血圧、糖尿病、メタボリックシンドロームに準じた複数のリスクを持つ者の割合が高かった。女性において、最も家計支出の高い群に比較した最も低い群のオッズ比は1.39から1.71だった。婚姻状態との関連では、女性では既婚者は未婚者よりリスク要因の割合が高くなっていたが、男性では逆の関係が認められた。この研究では、女性でのみ家計支出は循環器疾患のリスク要因と関連していた。社会経済的に好ましくない状況にある者ほど、循環器疾患になりやすくなる可能性が示唆されたが、性別や婚姻状態によりその関連性は異なった。健康づくりや循環器疾患の予防において社会経済的な要因にも考慮しなければならないことが示された。

キーワード：健康格差、社会経済的要因、家計支出、循環器疾患リスク、婚姻状態
